# Antinociceptive effects of caloric restriction on post-incisional pain in nonobese rats

**DOI:** 10.1038/s41598-017-01909-8

**Published:** 2017-05-11

**Authors:** Yue Liu, Yuan Ni, Wei Zhang, Yu-E Sun, Zhengliang Ma, Xiaoping Gu

**Affiliations:** Department of Anesthesiology, Affiliated Drum Tower Hospital of Medical School of Nanjing University, Nanjing, 210008 Jiangsu province China

## Abstract

Caloric restriction (CR) increases lifespan, retards physiological signs of aging, and delays a variety of diseases. Reduction of inflammatory response was proposed as one of the molecular mechanisms for how CR exerts beneficial effects. The present study investigated the effects of CR on postoperative pain in rats. Adult nonobese rats were divided into two dietary groups, an *ad libitum* fed group (AL) and a caloric restriction group (CR) that was provided with 60% of the food intake of AL rats. After 6 weeks, the effects of CR on pain behaviors and inflammation induced by plantar incision were examined. CR rats displayed significantly reduced nonevoked pain, mechanical allodynia and thermal hyperalgesia induced by incision, and showed decreased levels of pro-inflammatory cytokines in serum, peri-incisional skin tissue and ipsilateral spinal cord dorsal horn at 6 h and 24 h after incision. The analgesic efficiency of parecoxib and morphine, two agents widely used for the management of postoperative pain clinically, was reinforced by CR. Together, CR generates antinociceptive effects on postoperative incisional pain in rats, perhaps providing some improvement of QOL in patients with postoperative pain, and the beneficial effects may be attributable to the inhibition of excessive inflammation induced by surgical injury.

## Introduction

Caloric restriction (CR), defined as a dietary regimen low in total calories with maintenance of the essential composition of the diet at a normal level, such as vitamins and minerals, in the interest of avoiding malnutrition^1^, is a potent intervention which increases both average and maximum lifespan, retards physiological signs of aging, and decreases incidence and severity of age-related diseases, such as neoplasias, kidney disease, autoimmune disease, diabetes, in a variety of model organisms^2^. A recent multisite randomized clinical trial indicated that two years of 25% CR in healthy nonobese adults had positive effects and no negative effects on mood, sleep and quality of life (QOL)^3^.

A number of studies proposed a variety of molecular mechanisms for how CR exerts beneficial effects probably, for example, reduction of inflammatory response, up-regulation of endogenous and exogenous antioxidants, improvement of lipid metabolism, adjustment of neuroendocrine, and changes in apoptosis^4–6^. In the nervous system, CR regimen has been shown to be neuroprotective. Principally, CR delays neuroinflammation associated with aging^7^, attenuates pro-inflammatory pathways and enhances anti-inflammatory pathways in the hypothalamic^8^, regulates N-methyl-D-aspartate (NMDA) receptors and brain-derived neurotrophic factor (BDNF) expression in the hippocampus^9, 10^, and increases neuropeptide Y (NPY) levels in the hypothalamic^11^. These findings raise the possibility that more people, including obese and normal-weight people, might attempt to practice CR in an effort to increase lifespan and health span. However, the effects of CR on postoperative pain, which occurs after most surgeries and seriously influences patients’ QOL, are still unknown.

Approximately 40% of patients suffer moderate to severe pain during the postoperative period despite the aggressive use of available medications^12^. One of the fundamental causes of this pain involves the surgery-induced production and release of a variety of pro-inflammatory mediators in the wound site, blood and spinal cord, which initiates and maintains hyperalgesia^13–17^. However, there is no direct evidence currently from animal or human studies showing the effects and mechanisms of CR on postoperative incisional pain. Previous studies indicated that the prevalence of significant knee, hip, and back pain increases with increased levels of body weight among older Americans^18, 19^. Weight loss reduces pain and improves physical functioning in persons with symptomatic knee osteoarthritis^20^. CR can cause clear reductions in body weight and fat mass. Moreover, short term and intermittent food deprivation (IFD), another dietary regimen but inducing states physiologically different from CR, can generate antinociceptive effects in laboratory rodents^21, 22^. However, there were opposite results reported. Chronic food restriction has been found to alleviate the development of adjuvant arthritis in rats, but to increase the nociception in arthritic rats in the tail-flick test^23^. Moreover, acute food deprivation (fasting) enhanced the nociception in the formalin test in young female but not male rats^24^.

Considering that the various biochemical changes, especially systemic and local anti-inflammatory effects induced by CR may be also relevant to the transduction and regulation of incision-induced nociception, we hypothesized that CR exerts beneficial effects on postoperative incisional pain, and conducted the present study to probe into the potential mechanisms. In this study, daily calorie intake of adult nonobese rats was reduced by 40% for 6 weeks prior to classical hindpaw plantar incision to imitate postoperative pain. The effects of CR on pain behaviors, paw swelling, changes in cytokines, as well as the analgesic efficiency of opioids and Nonsteroidal anti-inflammatory drugs (NSAIDs) were investigated.

## Results

### Effects of caloric restriction on Body weight, Lee’s index and motor activity

**Figure 1:**
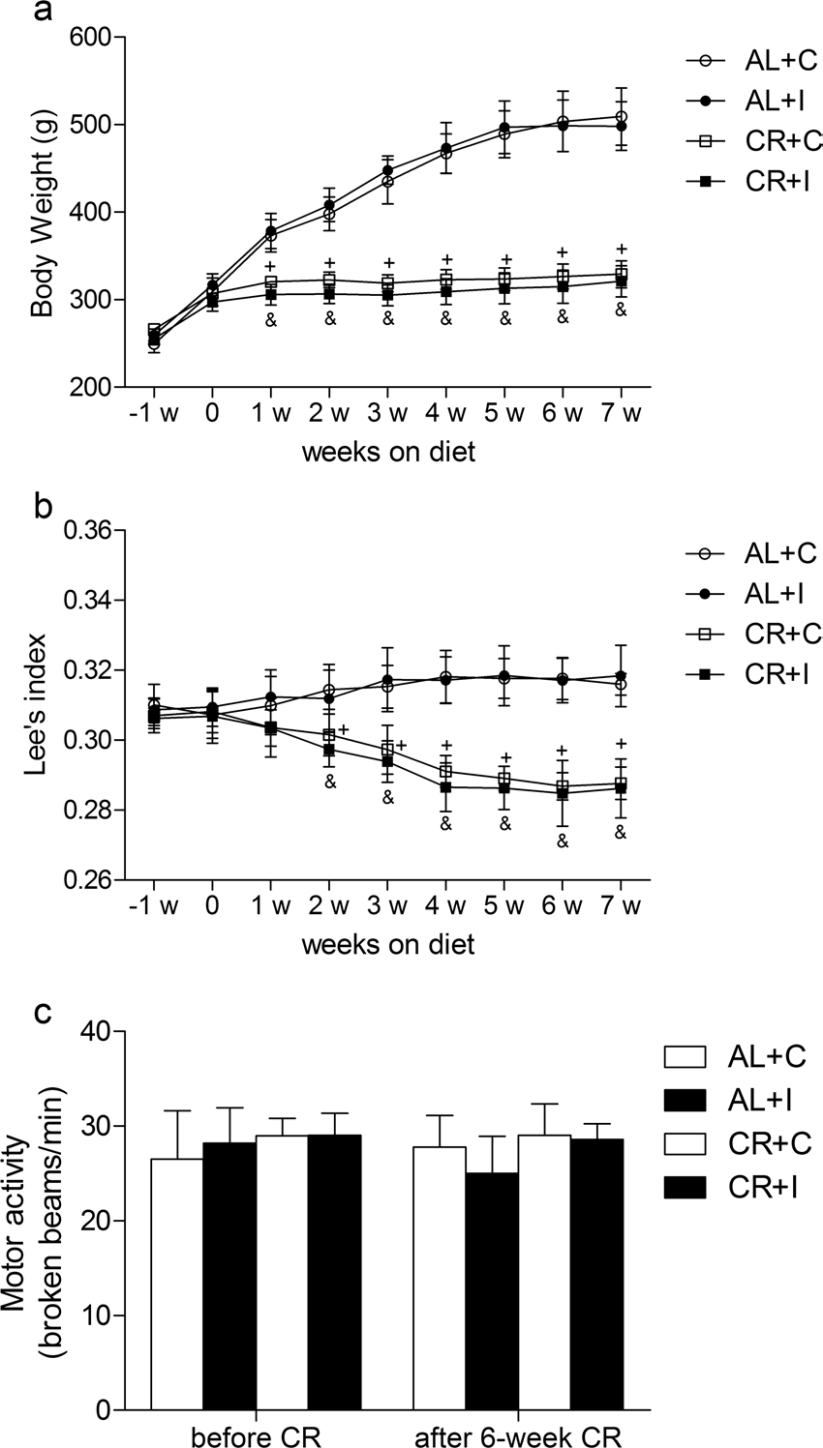
Effects of caloric restriction on Body weight, Lee’s index and motor activity. (**a**) Body weight (in grams) changes during the period of dietary treatment (n = 8). (**b**) Lee’s index changes during the period of dietary treatment (n = 8). (**c**) Locomotor activity in nonstressful conditions before and after a 6-week diet. Activity was indicated as broken beams per minute (n = 8). Values expressed as mean ± SD. AL+C: rats fed *ad libitum* received a sham procedure; AL+I: rats fed *ad libitum* received the incisional surgery on the right hindpaw; CR+C: rats fed a calorie-restricted diet received a sham procedure; CR+I: rats fed a calorie-restricted diet received the incisional surgery on the right hindpaw. Significant difference was revealed after two-way ANOVA (^+^P < 0.05 in comparison of group CR+C with group AL+C, ^&^p < 0.05 in comparison of group CR+I with group AL+I, Bonferroni post hoc tests).

Caloric restriction led to a significant decrease in body weight that stabilized eventually at about 60–70% of the value of AL rats (P < 0.001) (Fig. 1a). CR rats also had significant Lee’s index loss at the end of the 40% CR (P < 0.001) while AL rats had no significant change in Lee’s index, which reflects whether or not a rat is obese, and indicates the levels of obesity by dividing the cube root of the body weight by the naso-anal length. The Lee’s index in group CR was significantly lower from weeks 2 to 7 compared with that of group AL (P < 0.001) (Fig. 1b). Furthermore, the locomotor responses under nonstressful conditions were evaluated using a locomotor activity box. CR rats were indistinguishable from AL rats in motor activity before and after a 6-week diet (Fig. 1c).

### Caloric restriction attenuated nonevoked pain, mechanical allodynia, thermal hyperalgesia and hindpaw edema after surgical incision

**Figure 2:**
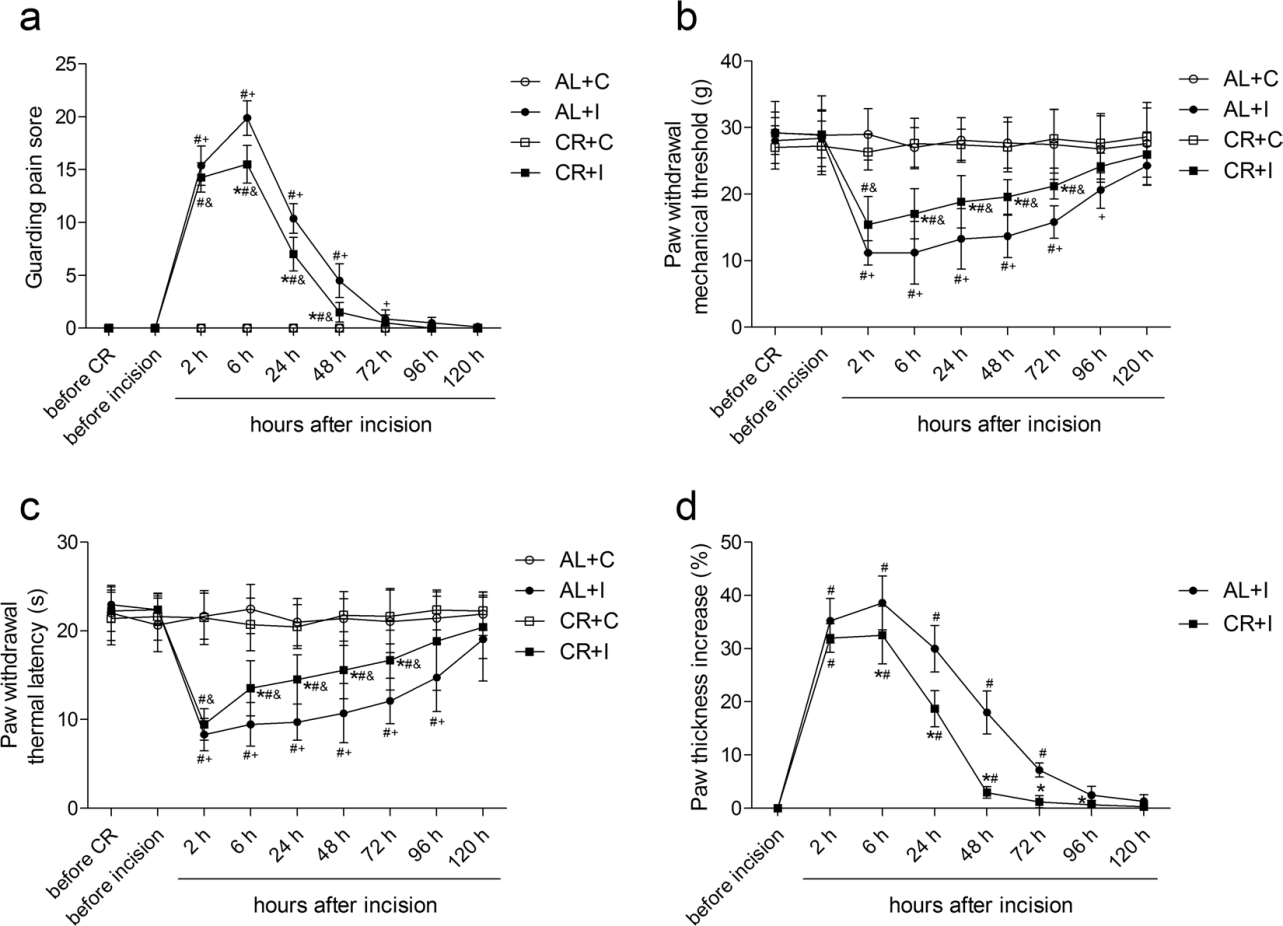
Caloric restriction attenuated nonevoked pain, mechanical allodynia, thermal hyperalgesia and hindpaw edema after surgical incision. (**a**) Caloric restriction attenuated incision-induced nonevoked pain presented as GPS of right hindpaw (n = 8). (**b**) Caloric restriction attenuated incision-induced mechanical allodynia presented as PWMT of right hindpaw (n = 8). (**c**) Caloric restriction attenuated incision-induced thermal hyperalgesia presented as PWTL of right hindpaw (n = 8). (**d**) Caloric restriction attenuated incision-induced right hindpaw edema presented as paw thickness increase (n = 8). Values expressed as mean ± SD. The treatment style was same as described in Fig. 1. Significant difference was revealed after two-way ANOVA (*P < 0.05 in comparison of group CR+I with group AL+I, ^#^P < 0.05 compared with baseline, ^+^P < 0.05 compared with group AL+C, ^&^p < 0.05 compared with group CR + C, Bonferroni post hoc tests).

The surgical incision was carried out on group CR+I and group AL+I after a 6-week diet. Spontaneous pain, mechanical allodynia and thermal hyperalgesia were assessed and presented as guarding pain score (GPS), paw withdrawal mechanical threshold (PWMT) and paw withdrawal thermal latency (PWTL). The baseline of GPS, PWMT and PWTL before the initiation of caloric restriction was not significantly different among groups. A 6-week caloric restriction did not change the basal pain perception in CR rats as compared with AL rats. As displayed in Fig. 2, hindpaw incision induced nonevoked pain, mechanical allodynia and thermal hyperalgesia in both groups CR+I and AL+I as compared with group CR+C and AL+C, respectively. Two-way ANOVA between group AL+I and group AL+C showed that there was a significant effect of group (GPS: F = 235.95, P < 0.001; PWMT: F = 15.61, P = 0.002; PWTL: F = 64.12, P < 0.001). Similarly, two-way ANOVA between group CR+I and group CR+C also showed a main effect of group (GPS: F = 229.11, P < 0.001; PWMT: F = 5.59, P = 0.024; PWTL: F = 99.89, P < 0.001). However, two-way ANOVA between group CR+I and group AL+I indicated significant main effects of caloric restriction (GPS: F = 15.25, P < 0.001; PWMT: F = 68.65, P < 0.001; PWTL: F = 27.43, P < 0.001). The GPS of rats subjected to caloric restriction plus incision were significantly decreased, PWMT and PWTL of rats subjected to caloric restriction plus incision were significantly increased than that of rats fed *ad libitum* subjected to incision (Fig. 2a–c). In addition, the GPS returned to baseline levels at 72 h, PWMT and PWTL returned to baseline levels at 96 h after surgery in group CR+I, while AL rats subjected to incision recovered at 96 h for GPS, at 120 h for PWMT and PWTL.

Edema is a common feature of tissue surrounding surgical wounds. Effects of caloric restriction on hindpaw edema in the incisional model were also observed. Paw thickness from dorsal side to ventral side was measured using a digital electronic bench micrometer. Because of the differences in the starting paw thickness between groups (AL+I starting thickness: 5.53 ± 0.26 mm, CR+I starting thickness: 4.91 ± 0.23 mm), we compared the increasing rate of the thickness measurements using the starting thickness of each rat as a reference (Fig. 2d). Paw incision significantly induced edema in a time dependent manner in both groups AL+I and CR+I. However, caloric restriction led to a statistically significant reduction in incision-induced paw thickness increase as compared with that of rats fed *ad libitum* after incision. Two-way ANOVA between group CR+I and group AL+I indicated a significant main effect of caloric restriction (F = 36.28, P < 0.001).

Because CR and AL rats were indistinguishable in locomotor activity, the observed reduction in pain behaviors evoked by incision appears to be attributable to caloric restriction-induced attenuation in this pain sensitization, rather than to a defect in motor functions in CR rats. In addition, caloric restriction accelerated the recovery from surgical pain.

### Effects of caloric restriction on the efficiency of analgesics after incision

**Figure 3:**
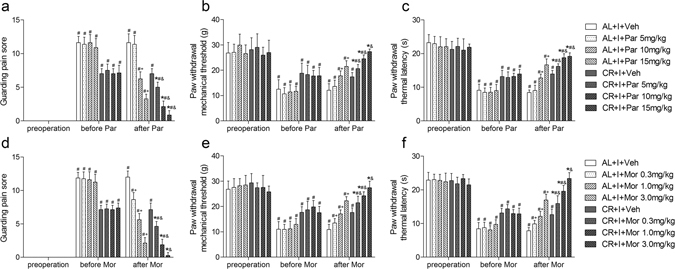
Effects of caloric restriction on the efficiency of analgesics after incision. A single intraperitoneal injection of parecoxib dose-dependently attenuated nonevoked pain (**a**), mechanical allodynia (**b**) and thermal hyperalgesia (**c**) (n = 8). A single subcutaneous injection of morphine dose-dependently attenuated nonevoked pain (**d**), mechanical allodynia (**e**) and thermal hyperalgesia (**f**) (n = 8). Pain behaviors were assessed before incision, before administration of analgesics on postoperative day 1 and at 1 h after administration. Values expressed as mean ± SD. AL+I+Par: AL+I rats received different doses of parecoxib (Par); CR+I+Par: CR+I rats received different doses of parecoxib; AL+I+Mor: AL+I rats received different doses of morphine (Mor); CR+I+Mor: CR+I rats received different doses of morphine; AL+I+Veh: AL+I rats received an equivalent volume of vehicle (saline); CR+I+Veh: CR+I rats received an equivalent volume of vehicle (saline). Significant difference was revealed after two-way ANOVA (*P < 0.05 in comparison of CR+I rats with AL+I rats received the identical dose of analgesic, ^#^P < 0.05 compared with baseline before incision, ^+^P < 0.05 compared with group AL+I+Veh, ^&^p < 0.05 compared with group CR+I+Veh, Bonferroni post hoc tests).

NSAIDs and opioids are widely used for the management of postoperative pain^25^. Here effects of caloric restriction on the analgesic efficiency of parecoxib and morphine, two well-known and widely used analgesic agents, were measured at 1 h after single intraperitoneal (i.p.) administration of parecoxib or subcutaneous (s.c.) administration of morphine on postoperative day 1 (Fig. 3). The doses of 5, 10, 15 mg/kg of parecoxib and 0.3, 1.0, 3.0 mg/kg of morphine both produced dose-dependent analgesic effects. Two-way ANOVA between group CR+I and group AL+I with equivalent dose of Parecoxib indicated a significant main effect of caloric restriction (GPS: all P < 0.001; PWMT: all P < 0.01; PWTL: all P < 0.001). Similarly, two-way ANOVA between group CR+I and group AL+I with equivalent dose of Morphine also indicated a significant main effect of caloric restriction (GPS: all P < 0.001; PWMT: all P < 0.001; PWTL: all P < 0.001).

The analgesic efficiency of Parecoxib and Morphine was represented as the maximal reversal of hyperalgesia, i.e. the percentage of the maximum possible effect (MPE). Parecoxib (15 mg/kg) produced a MPE of 88.62 ± 8.15%, 100 ± 35.55%, 65.63 ± 4.85% for GPS, PWMT, PWTL in CR rats, and 70.38 ± 3.58%, 65.91 ± 8.06%, 58.63 ± 7.49% for GPS, PWMT, PWTL in AL rats. Morphine (3.0 mg/kg) produced a MPE of 96.88 ± 5.79%, 100 ± 14.55%, 100 ± 6.34% for GPS, PWMT, PWTL in CR rats, and 81.51 ± 6.04%, 60.65 ± 8.40%, 57.50 ± 6.22% for GPS, PWMT, PWTL in AL rats, respectively. The MPE of parecoxib in CR rats was higher than that in AL rats, respectively (GPS: P < 0.001, PWMT: P = 0.004, PWTL: P = 0.043). Similarly, The MPE of morphine in CR rats was also higher than that in AL rats (all P < 0.001).

### Effects of caloric restriction on Inflammatory Biochemical Mediators in serum, peri-incisional skin tissue, and ipsilateral spinal cord dorsal horn after incision

Previous studies demonstrated that robust increases in the levels of several cytokines and chemokines play an important role in the development of hyperalgesia^16, 17^. Here effects of caloric restriction on the levels of inflammatory mediators in serum, peri-incisional skin tissue, and ipsilateral spinal cord dorsal horn before, 6 h, and 24 h after surgical incision were detected. There was no significant difference in the concentration of detected mediators in serum, peri-incisional skin tissue or spinal cord dorsal horn between group AL+I and group CR+I before incision.

**Figure 4:**
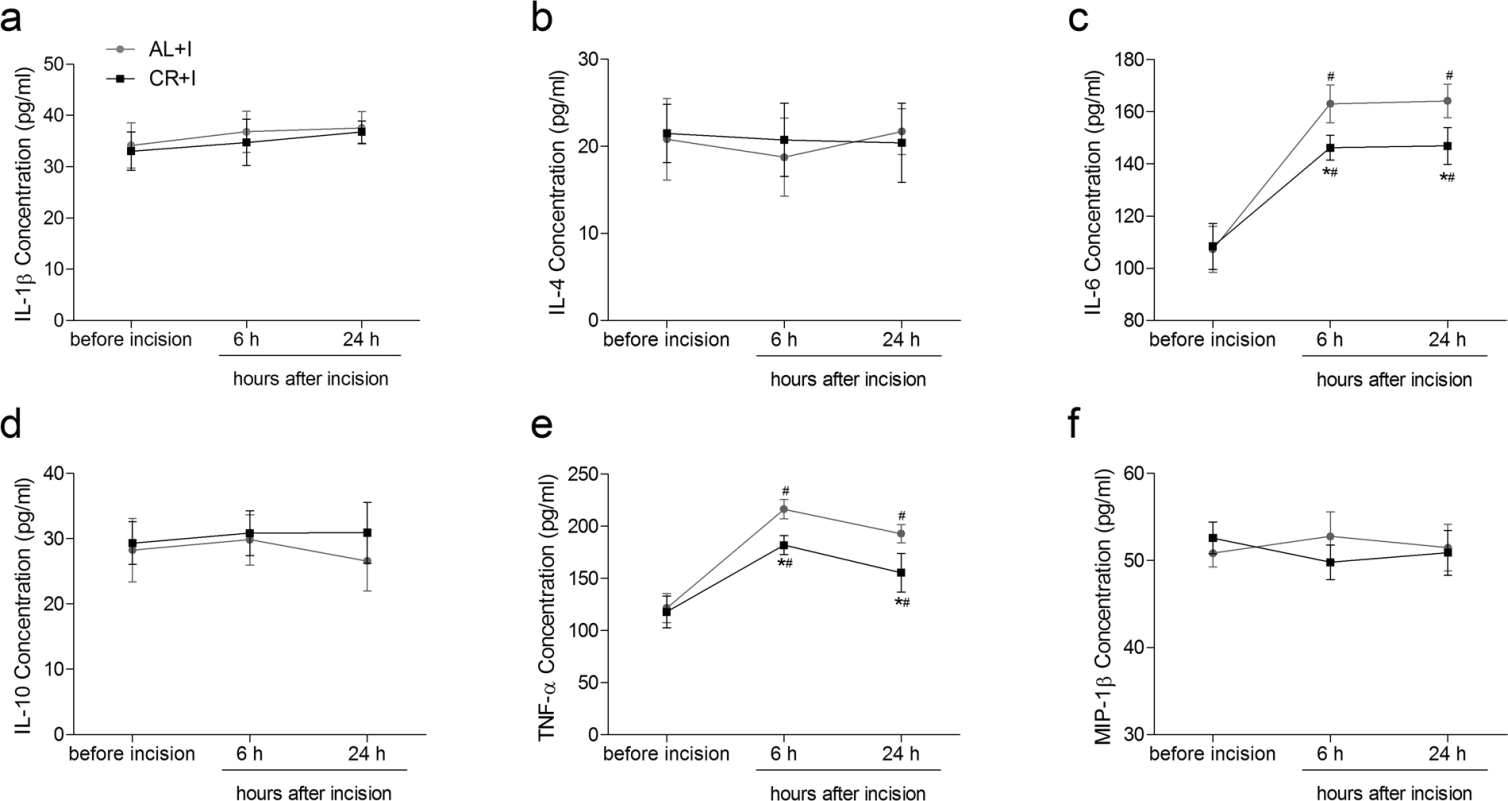
Effects of caloric restriction on pro-inflammatory and anti-inflammatory cytokines in serum after incision. Levels of IL-1β (**a**), IL-4 (**b**), IL-6 (**c**), IL-10 (**d**), TNF-α (**e**) and MIP-1β (**f**) in serum (pg/mL) measured before, 6 h, and 24 h after incisional surgery (n = 5). Values expressed as mean ± SD. The treatment style was same as described in Fig. 1. Significant difference was revealed after two-way ANOVA (*P < 0.05 in comparison of group CR+I with group AL+I, ^#^P < 0.05 compared with baseline, Bonferroni post hoc tests).

In serum, interleukin-6 (IL-6) and tumor necrosis factor-α (TNF-α) were significantly increased after surgical incision in group AL+I (all P < 0.01) and group CR+I (all P < 0.01). In both groups, IL-6 peaked at 6 h and levels were sustained for 24 hours, TNF-α was at its highest measured level at 6 h and decreased at 24 h. Two-way ANOVA showed significant main effects of caloric restriction (IL-6: F = 16.962, P < 0.01; TNF-α: F = 28.024, P < 0.01), time (IL-6: F = 139.184, P < 0.01; TNF-α: F = 96.886, P < 0.01), and interaction between time and CR (IL-6: F = 5.113, P = 0.014; TNF-α: F = 5.115, P = 0.013). Caloric restriction significantly decreased the measured levels of IL-6 (both P < 0.01) and TNF-α (both P < 0.01) at 6 h and 24 h after incision as compared with those of group AL+I. No changes over time were observed for IL-1β, IL-4, IL-10, or macrophage inflammatory protein-1β (MIP-1β) (Fig. 4).

**Figure 5:**
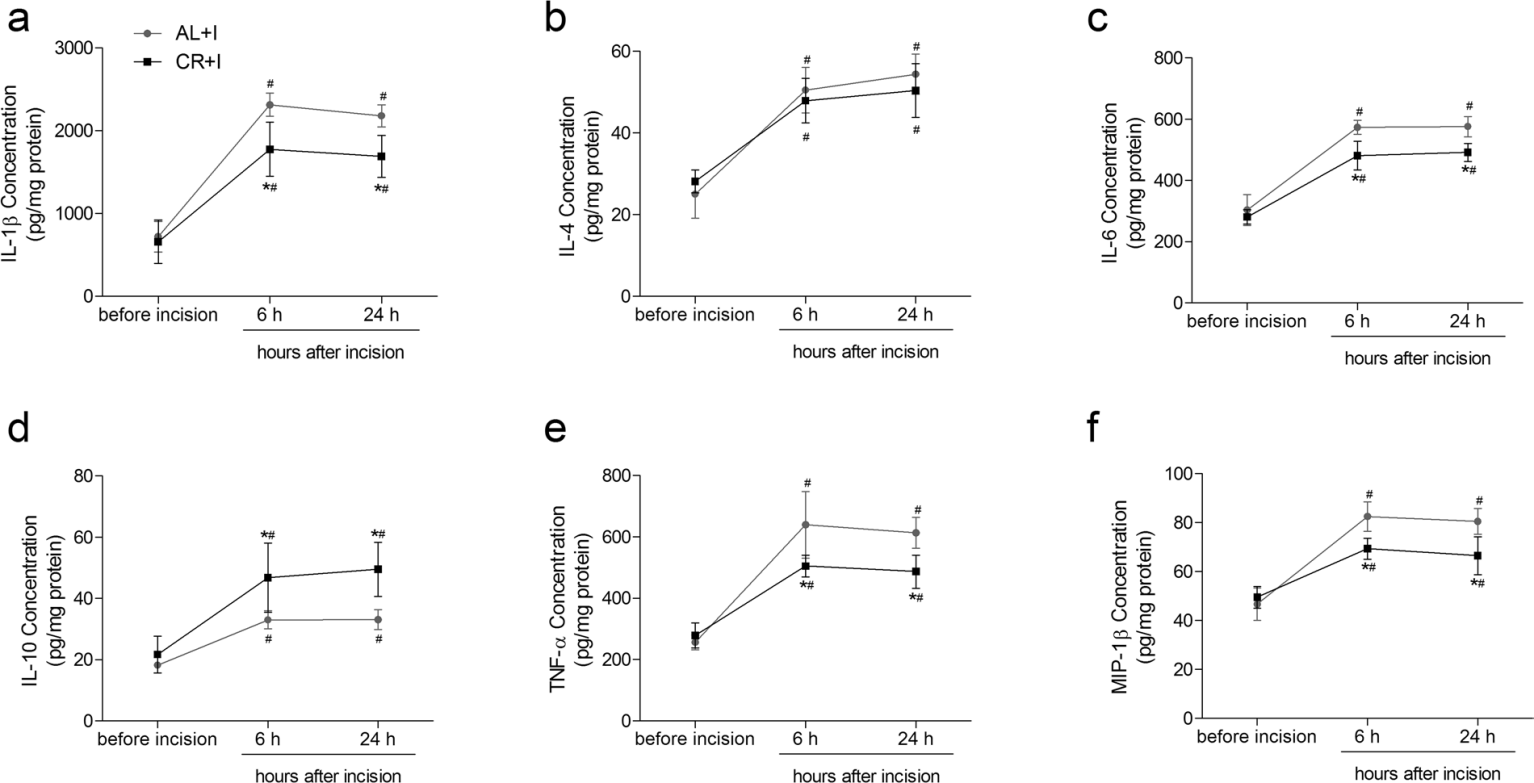
Effects of caloric restriction on pro-inflammatory and anti-inflammatory cytokines in peri-incisional skin tissue after incision. Levels of IL-1β (**a**), IL-4 (**b**), IL-6 (**c**), IL-10 (**d**), TNF-α (**e**) and MIP-1β (**f**) in peri-incisional skin tissue (pg/mg protein) measured before, 6 h, and 24 h after incisional surgery (n = 5). Values expressed as mean ± SD. The treatment style was same as described in Fig. 1. Significant difference was revealed after two-way ANOVA (*P < 0.05 in comparison of group CR+I with group AL+I, ^#^P < 0.05 compared with baseline, Bonferroni post hoc tests).

In peri-incisional skin tissue, significant increases over time were detected for IL-1β, IL-4, IL-6, IL-10, TNF-α and MIP-1β after incision in group AL+I (all P < 0.01) and group CR+I (all P < 0.01). They typically achieved maximum at 6 h and levels were sustained for 24 h. Two-way ANOVA showed significant main effects of caloric restriction (IL-1β: F = 19.017, P < 0.01; IL-6: F = 25.714, P < 0.01; IL-10: F = 21.637, P < 0.01; TNF-α: F = 13.683, P < 0.01; MIP-1β: F = 14.51, P < 0.01), time (IL-1β: F = 108.994, P < 0.01; IL-4: F = 69.268, P < 0.01; IL-6: F = 146.17, P < 0.01; IL-10: F = 32.876, P < 0.01; TNF-α: F = 82.988, P < 0.01; MIP-1β: F = 69.881, P < 0.01), and interaction between time and CR (TNF-α: F = 5.62, P = 0.01; MIP-1β: F = 6.627, P < 0.01). Caloric restriction significantly decreased the levels of IL-1β (both P < 0.01), IL-6 (both P < 0.01), TNF-α (both P < 0.01), MIP-1β (both P < 0.01), and increased the levels of IL-10 (both P < 0.01) at 6 h and 24 h after incision as compared with group AL+I (Fig. 5).

**Figure 6:**
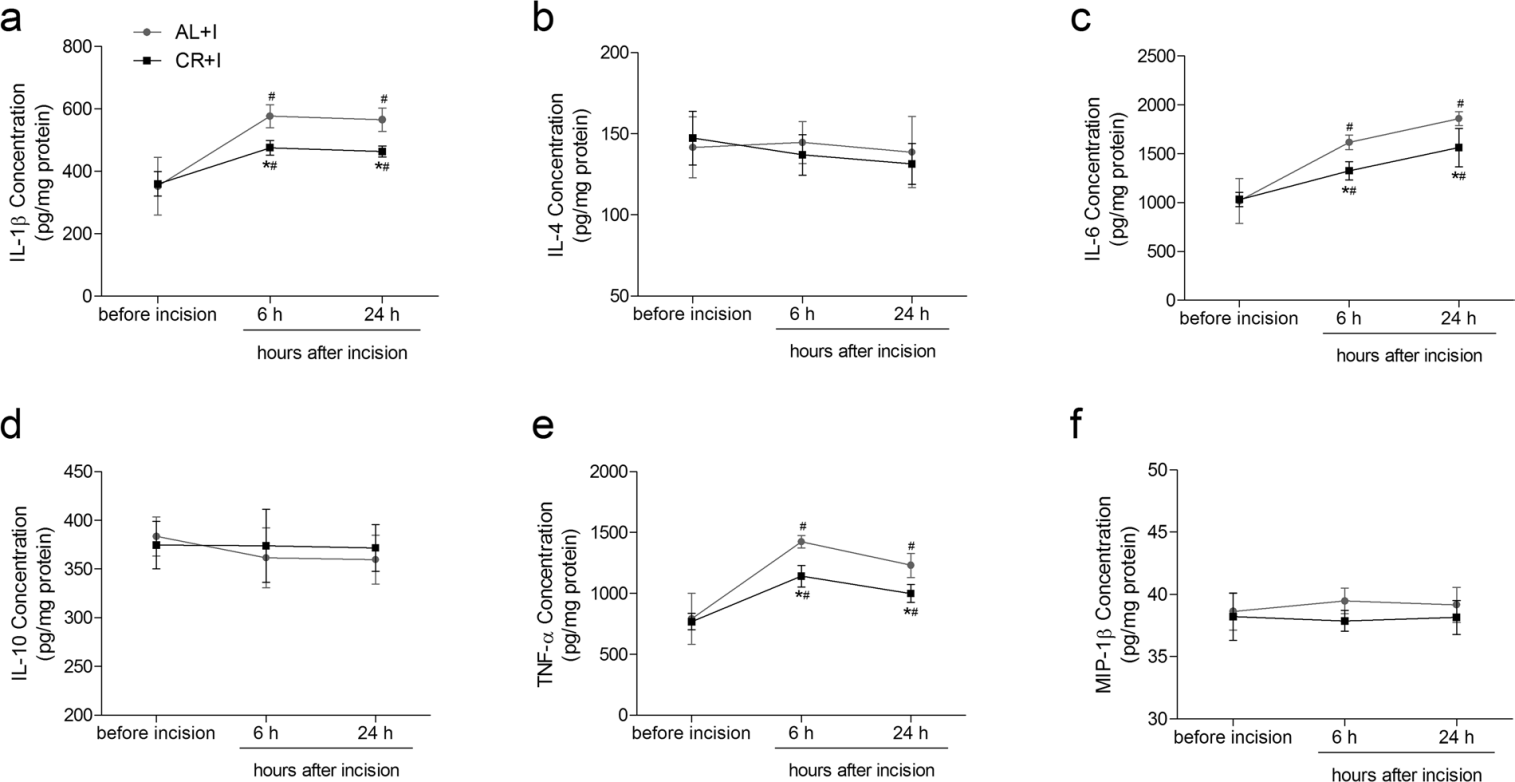
Effects of caloric restriction on pro-inflammatory and anti-inflammatory cytokines in ipsilateral spinal cord dorsal horn after incision. Levels of IL-1β (**a**), IL-4 (**b**), IL-6 (**c**), IL-10 (**d**), TNF-α (**e**) and MIP-1β (**f**) in ipsilateral spinal cord dorsal horn (pg/mg protein) measured before, 6 h, and 24 h after incisional surgery (n = 5). Values expressed as mean ± SD. The treatment style was same as described in Fig. 1. Significant difference was revealed after two-way ANOVA (*P < 0.05 in comparison of group CR+I with group AL+I, ^#^P < 0.05 compared with baseline, Bonferroni post hoc tests).

In ipsilateral lumbar spinal cord dorsal horn, the concentration of IL-1β, IL-6 and TNF-α increased significantly over the 24-hour time course after incision in group AL+I (all P < 0.01) and group CR+I (all P < 0.01). IL-1β was at its highest measured level at 6 h and remained at that level at 24 h, while IL-6 increased steadily over the 24-hour time course. In contrast, TNF-α peaked at 6 h and decreased at 24 h. Two-way ANOVA showed significant main effects of caloric restriction (IL-1β: F = 14.014, P < 0.01; IL-6: F = 14.343, P < 0.01; TNF-α: F = 19.514, P < 0.01), time (IL-1β: F = 39.406, P < 0.01; IL-6: F = 63.217, P < 0.01; TNF-α: F = 52.561, P < 0.01), and interaction between time and CR (IL-1β: F = 4.317, P = 0.025; IL-6: F = 4.157, P = 0.028; TNF-α: F = 3.778, P = 0.037). Caloric restriction significantly decreased the levels of IL-1β (both P < 0.01), IL-6 (both P < 0.01) and TNF-α (both P < 0.01) at 6 h and 24 h after incision as compared with those of group AL+I. IL-4, IL-10 and MIP-1β in spinal cord dorsal horn did not change significantly (Fig. 6).

## Discussion

Caloric restriction has some positive effects and no negative effects on QOL in both obese and nonobese adults^26^. However, little has been known about the relationship between caloric restriction and postoperative incisional pain. The major findings of the present study were as follows: (1) a 6-week caloric restriction regimen in nonobese adult rats attenuated nonevoked pain, mechanical allodynia and thermal hyperalgesia induced by incision; (2) caloric restriction reinforced the analgesic efficiency of parecoxib and morphine; (3) the attenuation of systemic and local inflammation could be important in the antinociceptive effects of CR on incisional pain.

The rat model of surgical incision on hindpaw was used to produce postoperative pain. One of the vital causes of this pain is the injury-induced production and release of a variety of inflammatory mediators in the tissue surrounding incision^14, 15^. Moreover, serum or plasma inflammatory mediators, which reflect the systemic response to surgical tissue injury, are also elevated after incision^16^. Both the local and systemic mediators exert influence on peripheral and spinal neuroinflammation, involving activation of spinal glia and increase in spinal cytokines, and are crucial for initiating and maintaining pain and hyperalgesia^13^. Experimental and clinical studies provided evidence for the role of pro-inflammatory cytokines, including IL-1β, IL-6, TNF-α, increased by incision in the development of hyperalgesia^16, 17, 27^. Specifically, IL-1β not only sensitizes sensory neurons to thermal stimuli directly^28^, but also causes hyperalgesia through inducing the expression or release of other cytokines including IL-6, TNF-α, MIP-1^29^, and pro-nociceptive compounds such as substance P^30^, prostaglandins, and nerve growth factor^31^. Knockout of IL-1 receptor or over-expression of the IL-1 receptor antagonists in mice reduced the mechanical hyperalgesia after incision^27^. In addition, anti-inflammatory cytokines, including IL-4 and IL-10, also altered by incision, have anti-hyperalgesic activity, mainly via limiting the production of pronociceptive cytokines^32^.

A clinical trial indicated that short-term preoperative dietary restriction reduced serum pro-inflammatory cytokine levels after surgery, and modified certain aspects of the postoperative acute phase response^33^. In rodent models of various diseases, caloric restriction significantly attenuated inflammatory response^34^. The present study showed that the measured cytokines increased over time in serum, peri-incisional skin tissue and ipsilateral spinal cord dorsal horn, accompanied by increased GPS and decreased PWMT and PWTL after incision in rats fed *ad libitum*. The typical release profiles of IL-1β, IL-4, IL-10 and MIP-1β with a peak level recorded at 6 h and a prolonged plateau, IL-6 with a gradual increase over time, and TNF-α which peaked early at 6 h and decreased within 24 h, were similar to that seen in previous studies^14, 16, 17^. The 6-week restriction of caloric intake decreased the levels of pain-related pro-inflammatory cytokines (IL-1β, IL-6, TNF-α, MIP-1β), increased the anti-inflammatory cytokine (IL-10), and significantly attenuated nonevoked pain, mechanical allodynia and thermal hyperalgesia induced by plantar incision.

NSAIDs and opioids are two of the most commonly employed classes of analgesics for the management of postoperative pain^25^. The present results indicated that caloric restriction increased the MPE, and reinforced the analgesic efficacy of both parecoxib (NSAID) and morphine (opioid) on postoperative day 1. Parecoxib inhibits the activity of Cyclooxygenase-2 (COX-2) and decreases the synthesis of prostanoids, thereby causing peripheral and central anti-inflammatory effects^35^. Morphine acts primarily via activation of mu-opioid receptor located in the central nervous system. However, it has recently been reported that morphine activates NOD-like receptor protein 3 (NLRP3) inflammasomes and associated release of IL-1β in spinal dorsal horn microglia, leading to neuroinflammation and paradoxical opposition of analgesia^36, 37^. Therefore, the reinforced analgesic efficacy may be, at least partly, due to the anti-inflammatory effects of caloric restriction. Considering that a relatively large dose of parecoxib or morphine may cause various adverse effects, caloric restriction, which may help to reduce postoperative analgesics consumption, can decrease the analgesics-associated adverse effects incidence and improve the quality of postoperative pain relief.

In addition, there are two limitations in the present study. Firstly, the rats used were young adult rats (8-week old), and the effects of CR on postoperative pain in middle-aged or old rats have not been investigated. Previous studies demonstrated that CR in the young adult life phase did not cause malnutrition or growth retardation, and the changes of behaviors were not the results of malnutrition or deficits in the nervous system^38, 39^. However, more researches are still needed to demonstrate whether or not it is comparable to the situation in humans. Secondly, the present results are descriptive. Nevertheless, previous studies indicated that CR attenuates neuroinflammation through up-regulating silent information regulator 1 (SIRT1) in cortex and hippocampus^40, 41^. SIRT1 deacetylates nuclear factor kappa B (NF-κB) p65 at lysine 310, and inhibits the activity of NF-κB, which facilitates the synthesis and release of a wide cascade of cytokines, including IL-1β, TNF-α, IL-6 and MIP-1^42^. The transcription of NLRP3, which plays an important role in cleaving the precursor pro-IL-1β to its mature form^43^, is also dependent on the acetylation and activation of NF-κB^44^. Therefore, the present results, in combination with previous studies, could be sufficient to draw the conclusion that CR attenuated postoperative pain and reinforced the analgesic efficacy probably via inhibiting inflammation. And our findings may have implications for the development of novel therapeutic strategies for postoperative pain. Moreover, some other mechanisms may also play an important role in the antinociceptive effects of CR. A reduction in food intake may generate antinociceptive signals in the central nervous system, involving mu-opioid receptors, kappa-opioid receptors and adrenocortical hormones, to promote a riskier behavior during prolonged food deprivation^21, 22^. In contrary, fasting can act as a physiological stimulus of the endogenous pain-modulation pathway involving the vagus nerve and gonadal hormones, and exert pro-nociceptive effects in the formalin test in female rats^24^. Further studies are needed to explore the underlying mechanisms.

It may be reasonable to conclude from our studies that by reducing the caloric intake regularly, pain responses in a rat model of incisional pain can be attenuated, and the analgesic efficacy of parecoxib and morphine was reinforced. These beneficial effects may be attributable to the inhibition of the excessive systemic and local inflammation induced by incision. These findings may highlight a new aspect of postoperative pain treatment. Considering that normal biological functions other than nociceptive processing appeared not to be disturbed in persons and animals on a CR diet as reported in previous studies, caloric restriction, which is noninvasive, inexpensive to practice, implementable in various settings, is likely to provide some improvement of QOL in persons who practice CR if postoperative pain occurs. More work is still needed to aid our understanding of the role of caloric restriction in pain modulation.

## Methods

### Animals

Adult male Sprague-Dawley rats, aged 7 weeks, weighing 240–260 g, were provided by the Laboratory Animal Center of DrumTower Hospital. All rats were housed in individual clear cages in a climate-controlled room (22 ± 2 °C). The humidity was between 40% and 50%, and 12-hour of light was given starting at 06:00 h. Access to food and water was *ad libitum* for all newly arrived rats for a week of acclimation prior to the commencement of experimental procedures. All experimental procedures were approved by the Institutional Animal Care and Use Committee in Nanjing University and were in accordance with the NIH guidelines for the care and use of laboratory animals (NIH Publication 86-23). Efforts were made to minimize rats’ suffering and to use the minimum number of rats necessary to obtain valid results.

### Dietary regimens

A standard regime was applied for caloric restriction^45^. Both control and restricted rats received the same commercial laboratory pellets (Xietong rodent diet, Jiangsu Xietong Feed Co., LTD, China). The group AL was fed *ad libitum*, and the daily food intake was consistent across the experimental period and ranged from 25–35 g per day. Group CR received 60% of the amount consumed by group AL. Vitamin and mineral mixtures (Jiangsu Xietong Feed Co., LTD, China) were added to the diet of group CR to make the content of micronutrient identical to group AL to avoid malnutrition but whose final caloric content was 40% lower. Food was weighed before it was placed in the cage’s food hopper and was provided to group CR daily, approximately 1 h before the lights-off period. The dietary regimens began after a week of acclimation (approximately 8 weeks of age), continued for 6 weeks before the surgery and behavioral testing, and were extended throughout the testing period. Water was available *ad libitum* to all groups. During this period, body weight and Lee’s index (cube root of body weight in grams divided by nose-to-anus length in cm) were evaluated.

### Incisional surgery

The incisional surgery was conducted as described previously^46^. Rats were anesthetized with inhalation of sevoflurane (3.5% for induction and 3.0% for maintenance) (Hengrui, Shanghai, China) via a nose mask. Before surgery, 5% povidone-iodine solution was used to sterilize the plantar surface of the operated right hindpaw. After placing the hindpaw through a hole in a sterile drape, an incision with a length of 1 cm was made starting at 0.5 cm from the proximal edge of the heel and extending towards the toes. The skin and fascia were cut through to expose the underlying plantar muscle. The plantar muscle was elevated and incised longitudinally, but the muscle origin and insertion remained intact. After hemostasis with gentle pressure, the skin was sutured using 5–0 nylon. The wound site was covered with antibiotic ointment. The control rats received a sham procedure, which consisted of administration with sevoflurane but without incision. All rats were allowed to recover in their cages after surgery.

### Assessment of locomotor activity

Locomotor responses under nonstressful conditions were assessed using a Plexiglas locomotor activity box (100 cm × 100 cm × 20 cm) in a darkened room^47^. 4.5 cm off the ground, eight infrared beams in an X-Y matrix, 20 cm apart, were counted when broken. Locomotor activity was indicated as broken beams per minute.

### Pain behavioral observation

All behavioral tests were carried out between 11:00-15:00 h, in order to minimize possible satiety effects of recent feeding and the potential reward of feeding soon after behavioral tests. Prior to each test, rats were allowed to acclimatize to the environment for 30 min. All behavioral responses were measured by the experimenters who were blind to the design of the study, the intervention on rats and the treatment groups. Pain behaviors were assessed 1 day before the commencement of dietary regimens, 1 day before the incisional surgery after 6-week caloric restriction, at 2 h, 6 h, 24 h, 48 h, 72 h, 96 h, 120 h after surgical incision, and at 1 h before analgesics administration, at 1 h after analgesics administration on the postoperative day 1 (n = 8 rats per group). Because of a relatively rapid onset of action of parecoxib and morphine, pain behaviors were measured at 1 h after administration. Moreover, because of a relatively short duration of action of morphine, nonevoked pain, mechanical allodynia and thermal hyperalgesia were measured in separate groups of rats receiving morphine respectively, i.e. only one pain behavior was measured in each group.

### Nonevoked pain

The GPS of the hindpaw was calculated on the basis of weight bearing to assess nonevoked pain behaviors according to a previously described method^48^. Unrestrained rats were placed on a stainless steel mesh floor (20 cm × 10 cm × 14 cm). The incised hindpaws were observed during a 1-min period repeated every 5 min for 1 h. Depending on the position in which the hindpaw was found during the 1-min scoring period, a score of 0, 1, or 2 was given. Full weight bearing on the paw was present if the wound was blanched or distorted by the mesh (score = 0). If the paw was completely off the mesh, a score of 2 was recorded. If the area of the wound touched the mesh gently without blanching or distorting, a score of 1 was recorded. The sum of 12 scores (0 to 24) for each hindpaw was obtained as the GPS.

### Mechanical allodynia

Mechanical allodynia was assessed using a Dynamic Plantar Aesthesiometer (No. 37450, Ugo Basile, Varese, Italy). Each rat was placed in a transparent Plexiglas compartments (20 cm × 10 cm × 14 cm) on a metal mesh floor. A metal wire was pressed vertically against the central plantar surface and the force was increased uniformly. A positive response was defined as paw flinching or withdrawal of the hindpaw. The test was repeated five times with a 5-min interval between each application of force. The PWMT was recorded as the tolerance level in grams and the mean PWMT of each hindpaw was calculated by averaging the latter three tests.

### Thermal hyperalgesia

Thermal hyperalgesia to radiant heat was determined using an automatic Plantar Test (Hargreaves Apparatus, No. 37370, Ugo Basile, Varese, Italy). Each rat was placed in a clear plastic cage on an elevated glass plate. The movable heat stimulator was moved to focus onto the central plantar surface of the hindpaw through the glass plate. The nociceptive end points in the radiant heat test were the characteristic lifting or licking of the hindpaw, and the time to the end point was considered the PWTL. A cutoff time of 25 seconds as used to avoid tissue damage. There were 5 trials per rat and 5-min intervals between trials. The mean PWTL was obtained from the latter 3 stimuli.

### Drugs preparation and injection

Parecoxib (Pharmacia and Upjohn Company, Kalamazoo, MI), a selective COX-2 inhibitor, was dissolved in 0.9% saline and intraperitoneally injected in doses of 5, 10, 15 mg/kg. Morphine (Northeast Pharmaceutical Company, China) was dissolved in 0.9% saline and subcutaneously injected in doses of 0.3, 1.0, 3.0 mg/kg. A single injection of parecoxib and morphine was performed in each group respectively on postoperative day 1. For vehicle treatment, an equivalent volume of saline was used. The dose was selected according to previous reports^35, 49^ and our preliminary experiments. The percent reversal of hyperalgesia for each rat was quantified as the percentage of the maximum possible effect (MPE) according to the following equation:

$$\begin{array}{ccc}{\rm{M}}{\rm{P}}{\rm{E}}{\rm{ \% }} & = & ({\rm{t}}{\rm{r}}{\rm{e}}{\rm{a}}{\rm{t}}{\rm{m}}{\rm{e}}{\rm{n}}{\rm{t}}{\rm{r}}{\rm{e}}{\rm{s}}{\rm{p}}{\rm{o}}{\rm{n}}{\rm{s}}{\rm{e}}-{\rm{p}}{\rm{o}}{\rm{s}}{\rm{t}}{\rm{o}}{\rm{p}}{\rm{e}}{\rm{r}}{\rm{a}}{\rm{t}}{\rm{i}}{\rm{v}}{\rm{e}}{\rm{r}}{\rm{e}}{\rm{s}}{\rm{p}}{\rm{o}}{\rm{n}}{\rm{s}}{\rm{e}})/\\  &  & ({\rm{p}}{\rm{r}}{\rm{e}}{\rm{o}}{\rm{p}}{\rm{e}}{\rm{r}}{\rm{a}}{\rm{t}}{\rm{i}}{\rm{v}}{\rm{e}}{\rm{r}}{\rm{e}}{\rm{s}}{\rm{p}}{\rm{o}}{\rm{n}}{\rm{s}}{\rm{e}}-{\rm{p}}{\rm{o}}{\rm{s}}{\rm{t}}{\rm{o}}{\rm{p}}{\rm{e}}{\rm{r}}{\rm{a}}{\rm{t}}{\rm{i}}{\rm{v}}{\rm{e}}{\rm{r}}{\rm{e}}{\rm{s}}{\rm{p}}{\rm{o}}{\rm{n}}{\rm{s}}{\rm{e}})\times 100{\rm{ \% }}.\end{array}$$

### Enzyme-linked immunosorbent assay (ELISA)

Blood samples, ipsilateral lumbar spinal cord dorsal parts and tissue samples around the wound were collected individually from rats before, 6 h, and 24 h after incisional surgery. Tissue samples were homogenized in 0.1 M Phosphate Buffered Saline (PBS, pH 7.4). The tissue supernatants containing proteins were collected via centrifugation at 12,000 rpm for 20 min at 4 °C, and stored as aliquots at −70 °C. The protein concentration was determined using the BCA method according to the kit’s instructions (Beyotime Biotechnology, Shanghai, China). Pro-inflammatory cytokines (IL-1β, IL-6, TNF-α) and anti-inflammatory cytokines (IL-4, IL-10) in serum or in supernatants were quantified using appropriate ELISA kits (4 A Biotech Co. Ltd, Beijing, China), MIP-1β was measured using ELISA kits from Abcam (Abcam, Cambridge, UK). All procedures were performed in accordance with the manufacturer’s instructions. Total protein levels were used as an internal control for the analysis of the tissues. Serum and tissue cytokine levels were expressed in pg/ml and pg/mg protein, respectively. The lowest limits of detection for rat IL-1β, IL-4, IL-6, IL-10, TNF-α and MIP-1β were 15, 7, 60, 15, 15 and 10 pg/ml respectively.

### Statistical Analysis

Data were expressed as the mean ± SD. SPSS 16.0 (SPSS Inc., Chicago, IL) was used to conduct all the statistical analyses. Rats were assigned to different treatment groups in a randomized manner. A two-way analysis of variance (ANOVA) was performed to determine the overall differences at each time point for body weight, Lee’s index, motor activity, pain behaviors and ELISA results among groups. As there were three independent variables (timepoints, dietary regimen, and incision vs control treatment) when comparing pain behaviors among all 4 groups, two groups having only the time and the dietary regimen as independent variables were included in such a two-way ANOVA. One-way ANOVA was used to determine differences in MPE between AL and CR rats. In both cases, when significant main effects were observed, Bonferroni post hoc tests were conducted to determine the source(s) of these differences. A P-value <0.05 was considered statistically significant.

## References

[1] Koubova J, Guarente L (2003). How does calorie restriction work?. Genes Dev.

[2] Mattison JA (2012). Impact of caloric restriction on health and survival in rhesus monkeys from the NIA study. Nature.

[3] Martin CK (2016). Effect of Calorie Restriction on mood, quality of life, Sleep, and Sexual Function in Healthy Nonobese Adults The CALERIE 2 Randomized Clinical Trial. JAMA Intern Med.

[4] Lopez-Lluch G (2006). Calorie restriction induces mitochondrial biogenesis and bioenergetic efficiency. Proc Natl Acad Sci USA.

[5] Rankin JW, Shute M, Heffron SP, Saker KE (2006). Energy restriction but not protein source affects antioxidant capacity in athletes. Free Radic Biol Med.

[6] Ugochukwu NH, Figgers CL (2007). Caloric restriction inhibits up-regulation of inflammatory cytokines and TNF-alpha, and activates IL-10 and haptoglobin in the plasma of streptozotocin-induced diabetic rats. J Nutr Biochem.

[7] MacDonald L, Radler M, Paolini AG, Kent S (2011). Calorie restriction attenuates LPS-induced sickness behavior and shifts hypothalamic signaling pathways to ananti-inflammatory bias. Am J Physiol Regul Integr Comp Physiol.

[8] MacDonald L, Radler M, Paolini AG, Kent S (2011). Calorie restriction attenuates LPS-induced sickness behavior and shifts hypothalamic signaling pathways to an anti-inflammatory bias. Am J Physiol Regul Integr Comp Physiol.

[9] Monti B, Virgili M, Contestabile A (2004). Alterations of markers related to synaptic function in aging rat brain, in normal conditions or under conditions of long-term dietary manipulation. Neurochem Int.

[10] Lee J, Duan W, Long JM, Ingram DK, Mattson MP (2000). Dietary restriction increases the number of newly generated neural cells, and induces BDNF expression, in the dentate gyrus of rats. J Mol Neurosci.

[11] Aveleira CA (2015). Neuropeptide Y stimulates autophagy in hypothalamic neurons. Proc Natl Acad Sci USA.

[12] Pyati S, Gan TJ (2007). Perioperative pain management. CNS Drugs.

[13] Fu D, Guo Q, Ai Y, Cai H, Yan J, Dai R (2006). Glial activation and segmental upregulation of interleukin-1beta (IL-1beta) in the rat spinal cord after surgical incision. Neurochem Res.

[14] Spofford CM, Brennan TJ (2012). Gene expression in skin, muscle, and dorsal root ganglion after plantar incision in the rat. Anesthesiology.

[15] Liang DY (2010). Caspase-1 modulates incisional sensitization and inflammation. Anesthesiology.

[16] Carvalho B, Clark DJ, Angst MS (2008). Local and systemic release of cytokines, nerve growth factor, prostaglandin E2, and substance P in incisional wounds and serum following cesarean delivery. J Pain.

[17] Buvanendran A (2006). Upregulation of prostaglandin E2 and interleukins in the central nervous system and peripheral tissue during and after surgery in humans. Anesthesiology.

[18] Häuser W, Schmutzer G, Brähler E, Schiltenwolf M, Hilbert A (2014). The impact of body weight and depression on low back pain in a representative population sample. Pain Med.

[19] Andersen RE, Crespo CJ, Bartlett SJ, Bathon JM, Fontaine KR (2003). Relationship between body weight gain and significant knee, hip, and back pain in older Americans. Obes Res.

[20] Riddle DL, Stratford PW (2013). Body weight changes and corresponding changes in pain and function in persons with symptomatic knee osteoarthritis: a cohort study. Arthritis Care Res (Hoboken).

[21] Hargraves WA, Hentall ID (2005). Analgesic effects of dietary caloric restriction in adult mice. Pain.

[22] de los Santos-Arteaga M, Sierra-Dominguez SA, Fontanella GH, Delgado-Garcia JM, Carrion AM (2003). Analgesia induced by dietary restriction is mediated by the kappa-opioid system. J Neurosci.

[23] Jurcovicova J (2001). Stress of chronic food restriction attenuates the development of adjuvant arthritis in male Long Evans rats. Clin Exp Rheumatol.

[24] Khasar SG, Reichling DB, Green PG, Isenberg WM, Levine JD (2003). Fasting is a physiological stimulus of vagus-mediated enhancement of nociception in the female rat. Neuroscience.

[25] White PF, Kehlet H (2010). Improving postoperative pain management: what are the unresolved issues?. Anesthesiology.

[26] Warkentin LM, Das D, Majumdar SR, Johnson JA, Padwal RS (2014). The effects of weight loss on health-related quality of life: systematic review and meta-analysis of randomized trials. Obes Rev.

[27] Wolf G, Livshits D, Beilin B, Yirmiya R, Shavit Y (2008). Interleukin-1 signaling is required for induction and maintenance of postoperative incisional pain: Genetic and pharmacological studies in mice. Brain Behav Immun.

[28] Obreja O, Rathee P, Lips KS, Distler C, Kress M (2002). IL-1 beta potentiates heat-activated currents in rat sensory neurons: Involvement of IL-1RI, tyrosine kinase, and protein kinase C. FASEB J.

[29] Dinarello CA (2005). Interleukin-1beta. Crit Care Med.

[30] Skoff AM, Zhao C, Adler JE (2009). Interleukin-1alpha regulates substance P expression and release in adult sensory neurons. Exp Neurol.

[31] Neeb L (2011). IL-1β stimulates COX-2 dependent PGE_2_ synthesis and CGRP release in rat trigeminal ganglia cells. PLoS One.

[32] Souza PP, Brechter AB, Reis RI, Costa CA, Lundberg P, Lerner UH (2013). IL-4 and IL-13 inhibit IL-1β and TNF-α induced kinin B1 and B2 receptors through a STAT6-dependent mechanism. Br J Pharmacol.

[33] van Ginhoven TM (2011). Dietary restriction modifies certain aspects of the postoperative acute phase response. J Surg Res.

[34] Starr ME, Steele AM, Cohen DA, Saito H (2016). Short-Term Dietary Restriction Rescues Mice From Lethal Abdominal Sepsis and Endotoxemia and Reduces the Inflammatory/Coagulant Potential of Adipose Tissue. Crit Care Med.

[35] Padi SS, Jain NK, Singh S, Kulkarni SK (2004). Pharmacological profile of parecoxib: a novel, potent injectable selective cyclooxygenase-2 inhibitor. Eur J Pharmacol.

[36] Grace PM (2016). Morphine paradoxically prolongs neuropathic pain in rats by amplifying spinal NLRP3 inflammasome activation. Proc Natl Acad Sci USA.

[37] Hutchinson MR (2010). Evidence that opioids may have toll-like receptor 4 and MD-2 effects. Brain Behav Immun.

[38] Levay EA, Govic A, Penman J, Paolini AG, Kent S (2007). Effects of adult-onset calorie restriction on anxiety-like behavior in rats. Physiol Behav.

[39] Cunha-Santos J, Duarte-Neves J, Carmona V, Guarente L, Pereira de Almeida L, Cavadas C (2016). Caloric restriction blocks neuropathology and motor deficits in Machado-Joseph disease mouse models through SIRT1 pathway. Nat Commun.

[40] Chen D (2008). The role of calorie restriction and SIRT1 in prion-mediated neurodegeneration. Exp Gerontol.

[41] Fusco S (2012). A role for neuronal cAMP responsive-element binding (CREB)-1 in brain responses to calorie restriction. Proc Natl Acad Sci USA.

[42] Shinozaki S (2014). Inflammatory stimuli induce inhibitory S-nitrosylation of the deacetylase SIRT1 to increase acetylation and activation of p53 and p65. Sci Signal.

[43] Wen H, Miao EA, Ting JP (2013). Mechanisms of NOD-like receptor-associated inflammasome activation. Immunity.

[44] Latz E, Xiao TS, Stutz A (2013). Activation and regulation of the inflammasomes. Nat Rev Immunol.

[45] Pugh TD, Klopp RG, Weindruch R (1999). Controlling caloric consumption: protocols for rodents and rhesus monkeys. Neurobiol Aging.

[46] Brennan TJ, Vandermeulen EP, Gebhart GF (1996). Characterization of a rat model of incisional pain. Pain.

[47] Koob AO, Cirillo J, Babbs CF (2006). A novel open field activity detector to determine spatial and temporal movement of laboratory animals after injury and disease. J Neurosci Meth.

[48] Zahn PK, Gysbers D, Brennan TJ (1997). Effect of systemic and intrathecal morphine in a rat model of postoperative pain. Anesthesiology.

[49] Whiteside GT (2004). Pharmacological characterisation of a rat model of incisional pain. Br J Pharmacol.

